# Long-Term Cardiorenal Benefits of Sodium-Glucose Cotransporter-2 Inhibitors in Patients with Type 2 Diabetes Mellitus: A Real-World Single-Center Experience

**DOI:** 10.3390/jcm14186365

**Published:** 2025-09-09

**Authors:** Adnan Agha, Bachar Afandi, Saeed AlKaabi, Naser Abdulla Naser Salem Alshkeili, Mohammed Ali Alsharoon Alshemeili, Mohammed Mohammed Al Ghaithi, Mohammad Mohammed Hareb Alsaadi, Juma Alkaabi

**Affiliations:** 1 Department of Internal Medicine, College of Medicine and Health Sciences, United Arab Emirates University, Al Ain P.O. Box 15551, United Arab Emirates; 201907445@uaeu.ac.ae (M.M.A.G.); 201902018@uaeu.ac.ae (M.M.H.A.); 2 Department of Internal Medicine, Tawam Hospital, Al Ain P.O. Box 15258, United Arab Emirates; bafandi@seha.ae (B.A.); 201602665@uaeu.ac.ae (S.A.); 201900976@uaeu.ac.ae (N.A.N.S.A.); 201704504@uaeu.ac.ae (M.A.A.A.)

**Keywords:** type 2 diabetes mellitus, sodium-glucose cotransporter-2 inhibitors, cardiorenal benefits, cardiovascular risk reduction, ASCVD risk score, empagliflozin, dapagliflozin, canagliflozin, United Arab Emirates

## Abstract

**Background:** The United Arab Emirates (UAE) faces a high burden of type 2 diabetes mellitus (T2DM) and its complications. While sodium-glucose cotransporter-2 inhibitors (SGLT2i) have demonstrated cardiorenal benefits in clinical trials, real-world evidence on their association with calculated cardiovascular risk in Middle Eastern populations remains limited. This study evaluated the long-term real-world outcomes associated with SGLT2i use in Emirati patients with T2DM. **Methods:** We conducted a retrospective observational study of patients with T2DM initiated on SGLT2i (empagliflozin, dapagliflozin, or canagliflozin) at Tawam Hospital, UAE, between 1 January 2018 and 31 December 2018. Patients were followed for up to 5 years. Primary outcomes included changes in glycated hemoglobin (HbA1c), estimated glomerular filtration rate (eGFR), and body mass index (BMI). A key secondary outcome was the change in 10-year atherosclerotic cardiovascular disease (ASCVD) risk, calculated using the ACC/AHA Pooled Cohort Equations. **Results:** We included 185 patients (mean age 57 ± 12 years, 56.2% female), with 107 (57.8%) receiving empagliflozin, 54 (29.2%) dapagliflozin, 11 (5.9%) canagliflozin, and 13 (7.0%) who switched between agents. Significant improvements were observed in HbA1c (8.7 ± 1.8% to 8.2 ± 1.9%, *p* < 0.001), while eGFR showed preservation of renal function with an annual decline of 1.1 mL/min/1.73 m^2^. Among 120 patients eligible for ASCVD risk assessment (excluding 65 with established cardiovascular disease), the mean 10-year ASCVD risk decreased from 22.3 ± 5.3% at baseline to 19.3 ± 4.9% at 5 years (absolute reduction −3.0%, 95% CI −2.4 to −3.6%, *p* < 0.001). Serious adverse events were rare, including acute kidney injury (1.1%) and fractures (1.6%). No episodes of diabetic ketoacidosis or severe hypoglycemia were observed. **Conclusions:** In this real-world cohort from the UAE, SGLT2 inhibitor use was associated with sustained glycemic control, preserved renal function, and lower calculated 10-year cardiovascular risk over 5 years. These observational findings, noted in the context of comprehensive risk factor management, support the potential benefits of SGLT2i in high-risk Middle Eastern patients with T2DM, though prospective controlled studies are needed to confirm causality.

## 1. Introduction

Type 2 diabetes mellitus (T2DM) represents a major public health challenge globally, with particularly high prevalence in the Middle East and North Africa (MENA) region [1]. The United Arab Emirates (UAE) has one of the world’s highest diabetes prevalence rates, affecting approximately 16.3% of the adult population, with projections suggesting this could reach 21.4% by 2030 [2]. This epidemic is driven by rapid urbanization, sedentary lifestyles, genetic predisposition, and dietary changes characteristic of the nutrition transition in Gulf countries [3,4].

The pathophysiology of T2DM involves multiple defects including insulin resistance, β-cell dysfunction, and increased hepatic glucose production [5]. Beyond glycemic control, patients with T2DM face substantially increased risks of cardiovascular disease (CVD) and chronic kidney disease (CKD), which are leading causes of morbidity and mortality [6]. In the UAE, diabetic nephropathy is the primary cause of end-stage renal disease, accounting for approximately 40% of dialysis cases, highlighting the urgent need for therapies that provide cardiorenal benefits [7].

Sodium-glucose cotransporter-2 (SGLT2) inhibitors represent a paradigm shift in T2DM management. By inhibiting glucose reabsorption in the proximal renal tubule, these agents lower plasma glucose independently of insulin secretion [8]. Landmark cardiovascular outcome trials including EMPA-REG OUTCOME (empagliflozin), CANVAS (canagliflozin), and DECLARE-TIMI 58 (dapagliflozin) demonstrated that SGLT2 inhibitors (SGLT2i) reduce major adverse cardiovascular events (MACE) in high-risk patients, and robustly reduce heart failure hospitalizations and progression of kidney disease in broader population [9,10,11].

The cardiorenal benefits of SGLT2is extend well beyond glucose lowering, driven by multiple pleiotropic mechanisms. These include hemodynamic effects through natriuresis and reduction in intraglomerular pressure, metabolic benefits including improved myocardial energetics and ketone body utilization, anti-inflammatory properties with reduction in inflammatory markers and oxidative stress, decreased arterial stiffness and improved endothelial function, and favorable effects on adipokine profiles and epicardial adipose tissue [12,13]. Building on this evidence of broad cardiovascular effects, recent evidence from a comprehensive meta-analysis suggests SGLT2 inhibitors may reduce the risk of atrial fibrillation, which is particularly relevant given that patients with T2DM have a 40% increased risk of developing atrial fibrillation and its associated complications including stroke and heart failure [14]. These diverse mechanisms have led to their recommendation as preferred agents in patients with T2DM and established atherosclerotic cardiovascular disease, heart failure, or CKD in international guidelines [15,16].

However, most evidence comes from clinical trials conducted predominantly in Western populations, with limited representation from the Middle East. Real-world data on SGLT2i effectiveness in Middle Eastern populations remain scarce [17]. Genetic, environmental, and lifestyle factors unique to the Emirati population may influence treatment responses [18]. The high prevalence of obesity (>30%), metabolic syndrome, and vitamin D deficiency in the UAE population may impact drug efficacy [19,20].

Understanding the real-world outcomes associated with SGLT2i use in this specific population is crucial for optimizing diabetes care strategies. This study aimed to evaluate the long-term associations between SGLT2i use and glycemic control, renal function preservation, and calculated cardiovascular risk in Emirati patients with T2DM in routine clinical practice.

## 2. Materials and Methods

### 2.1. Study Design and Setting

This retrospective observational cohort study was conducted at Tawam Hospital, a tertiary care facility serving as a major referral center in Al Ain, Abu Dhabi, UAE. The hospital provides comprehensive diabetes care through specialized endocrinology clinics. The study protocol received approval from the Tawam Human Research Ethics Committee (Reference: MF2058-2022-866, approved 22 September 2022). As a retrospective study using de-identified data, the requirement for informed consent was waived by the ethics committee.

### 2.2. Study Population

We screened electronic medical records to identify adult patients (≥18 years) with T2DM who were prescribed and initiated on any SGLT2i (canagliflozin, dapagliflozin, or empagliflozin) between 1 January 2018, and 31 December 2018.

### 2.3. Selection Criteria

Eligible participants were UAE nationals or residents aged 18 years or older with a confirmed diagnosis of T2DM for at least one year before initiating SGLT2i therapy. Inclusion required regular follow-up with documented baseline and 12-month post-treatment data, including a complete set of baseline laboratory parameters such as HbA1c, renal function, and a lipid profile. Patients were excluded if they had type 1 or secondary forms of diabetes, were receiving insulin therapy at the time of SGLT2i initiation, or had severe renal impairment (eGFR <45 mL/min/1.73 m^2^). Additional exclusion criteria comprised uncontrolled hyperglycemia (baseline HbA1c >12%), pregnancy or lactation, active malignancy, a life expectancy of less than one year, or incomplete medical records leading to loss to follow-up within 12 months.

### 2.4. Data Collection

Data were systematically extracted from electronic medical records (Oracle Health EHR, formerly Cerner Millennium) using standardized case report forms. Two independent reviewers performed data extraction with discrepancies resolved by a third reviewer. Collected variables included demographic and anthropometric data, clinical parameters, and laboratory values at baseline, 12 months, and 5 years post-SGLT2i initiation.

### 2.5. Adverse Event Assessment

Serious adverse events (SAEs) were defined according to ICH-GCP (International Council for Harmonisation and Good Clinical Practice) criteria as events resulting in death, life-threatening conditions, hospitalization, persistent disability, or requiring medical intervention to prevent permanent impairment. SAE determination was based on medical record review, though formal adjudication for drug causality was not performed given the retrospective nature of the study.

### 2.6. Cardiovascular Risk Assessment

The 10-year atherosclerotic cardiovascular disease (ASCVD) risk was calculated using the 2013 ACC/AHA (American College of Cardiology/American Heart Association) Pooled Cohort Equations for each eligible patient at baseline and 5-year follow-up. As validated risk equations for Middle Eastern populations are not available, we used the “White” category for calculations, acknowledging this as a significant limitation that may introduce systematic bias in risk estimation. Patients with established ASCVD at baseline were excluded from this analysis.

### 2.7. Statistical Analysis

Statistical analysis was performed using SPSS version 29.0 (IBM Corp., Armonk, NY, USA). Continuous variables were expressed as mean ± standard deviation (SD) with 95% confidence intervals where appropriate. Categorical variables were presented as frequencies and percentages.

For the primary analysis, paired *t*-tests compared baseline and post-treatment values for normally distributed continuous variables. Given multiple secondary outcomes, we applied Bonferroni correction with significance set at *p* < 0.01 for the five key secondary outcomes to control Type I error. Specifically, these five outcomes were the changes in glycated hemoglobin (HbA1c), estimated glomerular filtration rate (eGFR), body mass index (BMI), systolic blood pressure, and the 10-year ASCVD risk score. Between-group comparisons utilized one-way ANOVA with post hoc Bonferroni correction. Chi-square tests compared categorical variables.

Missing data were handled using multiple imputation for the primary analysis, with sensitivity analyses using complete case analysis. *p*-values for secondary outcomes should be considered exploratory given multiple testing.

## 3. Results

### 3.1. Patient Characteristics

**Figure 1:**
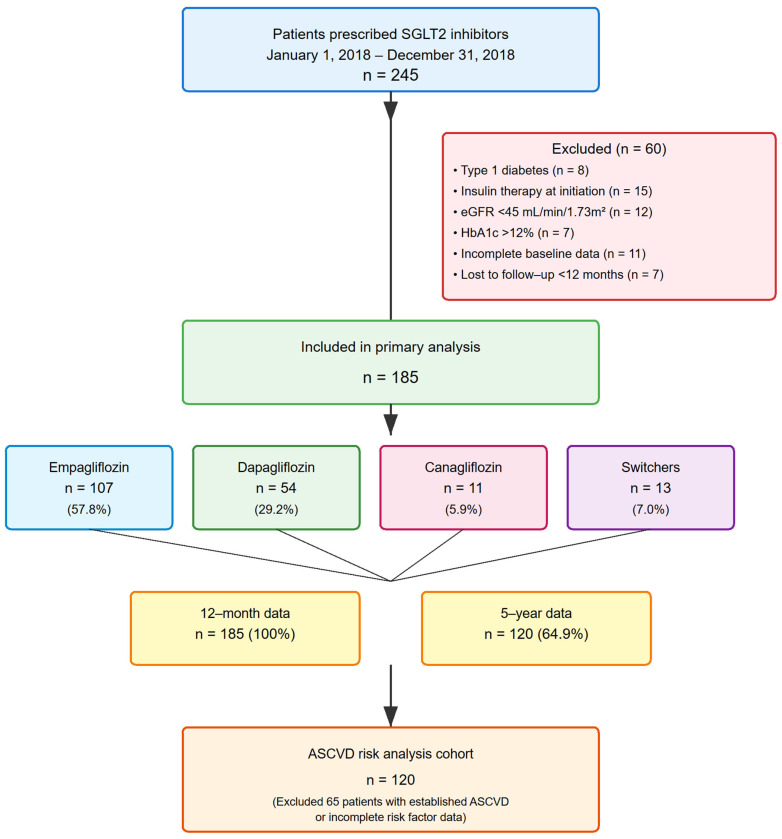
Patient flow diagram. Flow diagram showing patient selection and follow-up. Of 245 patients prescribed SGLT2 inhibitors between 1 January 2018 and 31 December 2018, 185 met inclusion criteria. Distribution: empagliflozin (n = 107, 57.8%), dapagliflozin (n = 54, 29.2%), canagliflozin (n = 11, 5.9%), and switchers (n = 13, 7.0%). Complete 5-year data available for 120 patients (64.9%).

A total of 245 patients initiated SGLT2i therapy during the study period. After applying exclusion criteria, 185 patients were included in the final analysis (Figure 1). The distribution by drug was: empagliflozin 107 (57.8%), dapagliflozin 54 (29.2%), canagliflozin 11 (5.9%), and 13 (7.0%) patients who switched between SGLT2i agents during follow-up.

The mean age at SGLT2i initiation was 57.0 ± 11.8 years, with 104 (56.2%) female patients. The mean diabetes duration was 8.2 ± 5.3 years. Baseline characteristics were generally well-balanced across treatment groups (Table 1). It is noted, however, that the canagliflozin group had a significantly higher mean baseline HbA1c (9.6 ± 1.9%) compared to the other groups (*p* = 0.042), a factor that should be considered given the small size of this subgroup. Complete 5-year follow-up data were available for 120 patients (64.9%). Comparison of baseline characteristics between patients with and without complete follow-up showed no significant differences (Supplementary Table S1).

### 3.2. Glycemic Control

HbA1c decreased significantly from baseline 8.7 ± 1.8% to 8.2 ± 1.9% at 12 months (mean reduction −0.5%, 95% CI −0.3 to −0.7%, *p* < 0.001). This improvement was sustained through 5 years of follow-up in patients with available data (n = 120), with mean HbA1c of 8.2 ± 1.8% at 5 years. The proportion of patients achieving HbA1c <7% increased from 18.4% at baseline to 31.9% at 12 months (*p* < 0.001).

**Figure 2:**
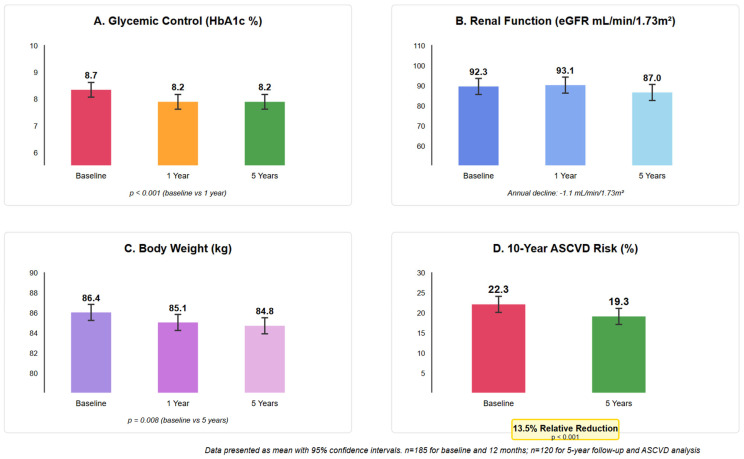
Key clinical outcomes over 5 years of SGLT2 inhibitor therapy. Changes in (**A**) HbA1c, (**B**) eGFR, (**C**) body weight, and (**D**) 10-year ASCVD risk. Data shown as mean ± SD. HbA1c decreased from 8.7% to 8.2% (*p* < 0.001). eGFR showed annual decline of 1.1 mL/min/1.73 m^2^. Weight decreased from 86.4 to 84.8 kg (*p* = 0.008). ASCVD risk decreased from 22.3% to 19.3% (13.5% relative reduction, *p* < 0.001).

See Figure 2 for details.

### 3.3. Renal Function

eGFR remained stable from 92.3 ± 22.1 to 93.1 ± 21.9 mL/min/1.73 m^2^ at 12 months (*p* = 0.32), indicating initial preservation of renal function. At 5 years, the mean eGFR was 87.0 ± 23.8 mL/min/1.73 m^2^, representing an annual decline of 1.1 mL/min/1.73 m^2^, which is numerically lower than historical reports of 1.5–2.5 mL/min/1.73 m^2^ annual decline in patients with T2DM [21], though direct comparison is limited by the lack of a control group.

### 3.4. Weight and BMI Changes

**Figure 3:**
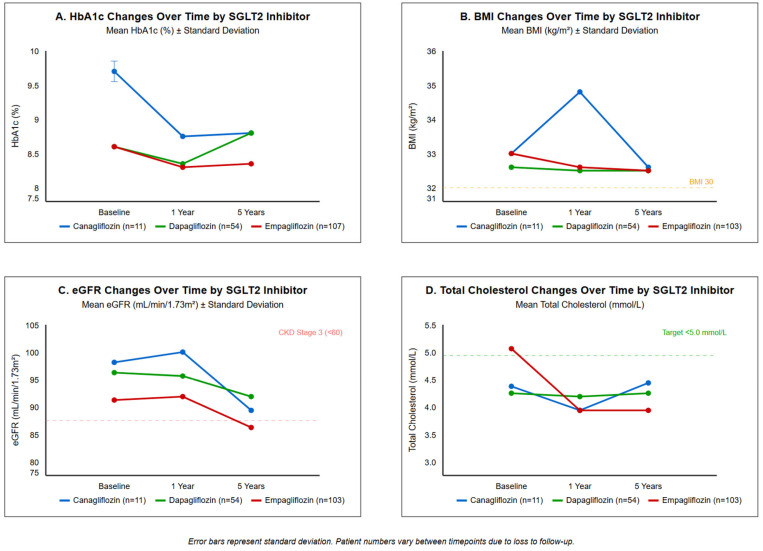
Longitudinal changes in metabolic and renal parameters by SGLT2 inhibitor type. Trajectories over 5 years for (**A**) HbA1c, (**B**) BMI, (**C**) eGFR, and (**D**) total cholesterol by drug type. Data shown as mean ± SD. Canagliflozin (n = 11), dapagliflozin (n = 54), empagliflozin (n = 107). Error bars represent standard deviation.

Mean weight decreased modestly from 86.4 ± 18.2 kg at baseline to 85.1 ± 17.9 kg at 12 months (mean reduction −1.3 kg, 95% CI −0.5 to −2.1 kg, *p* = 0.01). BMI decreased from 33.1 ± 6.7 to 32.7 ± 6.8 kg/m^2^ (*p* = 0.011). While statistically significant, these changes represent modest clinical effects. See Figure 3 for longitudinal changes in metabolic and renal parameters.

### 3.5. Cardiovascular Risk Assessment

**Figure 4:**
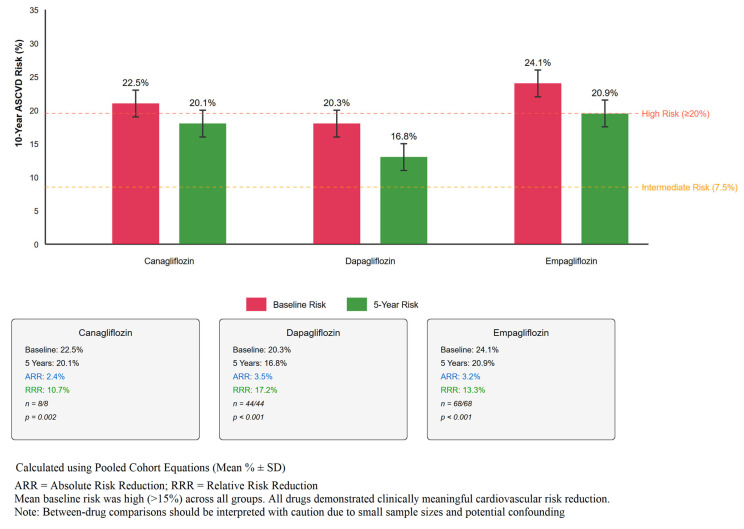
Distribution of 10-year ASCVD risk scores before and after 5 years of SGLT2 inhibitor therapy. Comparison of baseline and 5-year ASCVD risk by drug type. Mean ± SD shown with absolute and relative risk reduction. Canagliflozin: 22.5% to 20.1% (ARR 2.4%, RRR 10.7%, n = 8/8). Dapagliflozin: 20.3% to 16.8% (ARR 3.5%, RRR 17.2%, n = 44/44). Empagliflozin: 24.1% to 20.9% (ARR 3.2%, RRR 13.3%, n = 68/68).

Among the 185 patients, 120 had complete data for ASCVD risk calculation at both baseline and 5-year follow-up (65 were excluded due to established ASCVD at baseline). The mean 10-year ASCVD risk decreased from 22.3 ± 5.3% at baseline to 19.3 ± 4.9% at 5 years (absolute reduction −3.0%, 95% CI −2.4 to −3.6%, *p* < 0.001), representing a 13.5% relative reduction. However, this reduction occurred in the context of substantial treatment intensification, with statin use increasing from 71.4% to 78.3% and ACE inhibitor/ARB use from 63.8% to 69.2% over the study period. These improvements in cardiovascular risk profiles therefore reflect comprehensive risk factor management rather than SGLT2i effects alone. See Figure 4 for details.

### 3.6. Concomitant Medication Changes

Over the 5-year follow-up period, there was notable intensification of cardiovascular risk factor management. Statin use increased from 132 (71.4%) to 145 (78.3%) patients, ACE inhibitor or ARB use increased from 118 (63.8%) to 128 (69.2%), and antiplatelet therapy use increased from 89 (48.1%) to 102 (55.1%). These changes in concomitant therapy likely contributed to the observed improvements in cardiovascular risk profiles.

### 3.7. Safety Outcomes

SGLT2i therapy was generally well-tolerated (Table 2). Genital mycotic infections occurred in 16 patients (8.6%), with higher incidence in females (12.9% vs. 3.7%, *p* = 0.04). Urinary tract infections were reported in 21 patients (11.4%). Serious adverse events were rare: acute kidney injury occurred in 2 patients (1.1%) and fractures in 3 patients (1.6%). No episodes of diabetic ketoacidosis or severe hypoglycemia requiring hospitalization were documented. Seven patients (3.8%) discontinued therapy, primarily due to cost (n = 5) rather than adverse events (n = 2).

### 3.8. Outcomes in Patients Who Switched Between SGLT2 Inhibitors

The 13 patients who switched between SGLT2i agents during follow-up showed comparable outcomes to the overall cohort, with HbA1c reduction from 8.8 ± 1.7% to 8.3 ± 1.8% and preserved renal function (Supplementary Table S2).

## 4. Discussion

This real-world study provides important observational evidence on the long-term outcomes associated with SGLT2i use in a Middle Eastern population with T2DM. Our findings demonstrate associations with sustained glycemic control, preservation of renal function, and lower calculated cardiovascular risk over 5 years of follow-up. However, these associations must be interpreted cautiously given the observational design and lack of a control group.

### 4.1. Glycemic Efficacy in Real-World Settings

The HbA1c reduction of 0.5% observed in our cohort is modest compared to the 0.7–1.0% reductions reported in randomized controlled trials [9,10,11]. This efficacy-effectiveness gap is well-recognized in real-world diabetes studies and likely reflects several factors [22]. Our patients had a lower baseline HbA1c of 8.7% compared to >8.0% in most trials, real-world adherence is typically lower than in clinical trials, trial participants receive more intensive lifestyle interventions and monitoring, and our cohort had high rates of obesity (mean BMI >33 kg/m^2^) and long diabetes duration which may influence treatment response.

Despite the modest HbA1c reduction, the proportion achieving target HbA1c <7% increased significantly, and glycemic control was sustained over 5 years, contrasting with the typical progressive deterioration seen with other antidiabetic agents [23]. However, without a control group, we cannot definitively attribute these changes to SGLT2i therapy alone.

### 4.2. Cardiovascular Risk Assessment: Interpretation and Limitations

A key finding of our study is the observed 13.5% relative reduction in calculated 10-year ASCVD risk. However, this finding requires careful interpretation. The ASCVD risk reduction occurred in the context of overall treatment intensification, with increased use of statins and ACE inhibitors/ARBs over the study period. The observed risk reduction cannot be attributed to SGLT2i therapy alone but rather represents the cumulative effect of comprehensive diabetes and cardiovascular risk management, including the documented intensification of statin and ACE inhibitor/ARB therapy.

Furthermore, the use of the ACC/AHA Pooled Cohort Equations, which were validated in White and African American populations, represents a significant limitation. The lack of validated risk equations for Middle Eastern populations means our risk estimates may be systematically biased. Development of population-specific risk assessment tools is urgently needed for accurate cardiovascular risk stratification in this region.

### 4.3. Renal Outcomes in Context

The observed annual eGFR decline of 1.1 mL/min/1.73 m^2^ is numerically lower than historical reports of 1.5–2.5 mL/min/1.73 m^2^ in patients with T2DM [21]. While this suggests potential renal benefits, the absence of a matched control group prevents definitive conclusions about the protective effects of SGLT2i therapy. The initial stabilization of renal function at one year is consistent with the known hemodynamic effects of SGLT2is, though long-term benefits require confirmation in controlled studies.

### 4.4. Population-Specific Considerations

The effectiveness of SGLT2i in this cohort must be considered within the context of factors unique to the UAE population. High ambient temperatures could theoretically increase dehydration risk, though we observed no cases of severe volume depletion. Cultural dietary patterns and high rates of vitamin D deficiency may influence drug response. The high baseline cardiovascular risk in our population (mean ASCVD risk >22%) underscores the importance of aggressive risk factor modification in this setting.

### 4.5. Clinical Implications

Our observational findings, while not establishing causality, suggest potential benefits of SGLT2i therapy in Middle Eastern patients with T2DM. The observed associations with glycemic control, renal function preservation, and cardiovascular risk profiles support consideration of these agents in high-risk patients. However, clinicians should recognize that the benefits observed in our study likely reflect comprehensive diabetes care rather than SGLT2i effects alone.

### 4.6. Limitations

This study has several important limitations that must be acknowledged. First, the retrospective, single-center, observational design limits causal inference and generalizability. The observed associations may be influenced by unmeasured confounding factors. Second, only 65% of patients had complete 5-year follow-up data, introducing potential attrition bias, though baseline characteristics were similar between completers and non-completers. Third, the exclusion of patients with insulin use and eGFR <45 mL/min/1.73 m^2^ restricts generalizability to broader T2DM populations who may particularly benefit from SGLT2i therapy based on recent trials. Conversely, this focus provides specific real-world insights into the use of SGLT2 inhibitors as a foundational therapy in patients with relatively preserved renal function, a common and important scenario in routine clinical practice.

Fourth, the lack of a control group prevents direct attribution of observed changes to SGLT2i therapy. Historical comparisons are indirect and subject to bias. Fifth, we used calculated cardiovascular risk scores rather than actual cardiovascular outcomes, and the ASCVD equations may not accurately estimate risk in Middle Eastern populations. Sixth, we could not assess medication adherence or account for all changes in concomitant therapy over the 5-year period. Finally, the small sample size for canagliflozin (n = 11) and the imbalanced group sizes prevent meaningful between-drug comparisons.

## 5. Conclusions

In this real-world cohort of Emirati patients with T2DM, SGLT2 inhibitor use was associated with sustained glycemic control, preserved renal function, and lower calculated cardiovascular risk over 5 years. While these observational findings are encouraging and suggest potential benefits of SGLT2i therapy in Middle Eastern populations, they cannot establish causality. The observed improvements in cardiovascular risk and renal function preservation occurred in the context of comprehensive risk factor management, including intensification of cardioprotective medications, and cannot be attributed to SGLT2i therapy alone. Prospective controlled studies with actual cardiovascular and renal outcomes are needed to confirm the benefits of SGLT2i therapy in Middle Eastern populations. Healthcare systems in the region should consider these agents as part of comprehensive diabetes management strategies, particularly for high-risk patients, while recognizing the need for population-specific outcome studies.

## Figures and Tables

**Table 1:** Baseline characteristics of patients with type 2 diabetes mellitus by sodium-glucose cotransporter-2 inhibitor type.

Characteristic	Total (n = 185)	Canagliflozin (n = 11)	Dapagliflozin (n = 54)	Empagliflozin (n = 107)	Switchers (n = 13)	*p*-Value
Demographics						
Age (years)	57.0 ± 11.8	53.0 ± 17.0	56.0 ± 9.0	58.0 ± 12.0	56.5 ± 10.2	0.421
Female, n (%)	104 (56.2)	7 (63.6)	30 (55.6)	64 (59.8)	3 (23.1)	0.148
Diabetes duration (years)	8.2 ± 5.3	7.8 ± 4.9	8.1 ± 5.2	8.4 ± 5.5	8.0 ± 5.1	0.856
Clinical parameters						
Weight (kg)	86.4 ± 18.2	88.7 ± 16.3	85.2 ± 17.8	86.9 ± 18.7	85.1 ± 17.9	0.742
BMI (kg/m^2^)	33.1 ± 6.7	33.2 ± 4.4	32.3 ± 5.9	33.2 ± 7.1	32.8 ± 6.2	0.635
Systolic BP (mmHg)	132.5 ± 16.2	130.2 ± 14.8	131.8 ± 15.9	133.4 ± 16.7	132.1 ± 15.8	0.684
Diastolic BP (mmHg)	78.3 ± 9.8	77.5 ± 8.9	77.9 ± 9.6	78.7 ± 10.1	78.0 ± 9.5	0.821
Laboratory values						
HbA1c (%)	8.7 ± 1.8	9.6 ± 1.9	8.5 ± 1.6	8.5 ± 1.9	8.8 ± 1.7	0.042
FPG (mmol/L)	9.8 ± 3.2	10.9 ± 3.5	9.5 ± 3.0	9.7 ± 3.2	9.9 ± 3.1	0.238
eGFR (mL/min/1.73 m^2^)	92.3 ± 22.1	98.5 ± 23.2	97.1 ± 16.1	90.4 ± 23.1	91.2 ± 21.8	0.156
Total cholesterol (mmol/L)	4.5 ± 1.2	4.4 ± 0.9	4.3 ± 1.1	4.8 ± 1.3	4.5 ± 1.1	0.089
Comorbidities, n (%)						
Hypertension	126 (68.1)	7 (63.6)	35 (64.8)	75 (70.1)	9 (69.2)	0.684
Dyslipidemia	143 (77.3)	8 (72.7)	40 (74.1)	85 (79.4)	10 (76.9)	0.625
Current smoker	19 (10.3)	1 (9.1)	5 (9.3)	12 (11.2)	1 (7.7)	0.891
Concomitant medications, n (%)						
Metformin	172 (93.0)	10 (90.9)	50 (92.6)	100 (93.5)	12 (92.3)	0.912
Sulfonylurea	68 (36.8)	4 (36.4)	19 (35.2)	40 (37.4)	5 (38.5)	0.954
ACE-I/ARB	118 (63.8)	6 (54.5)	33 (61.1)	71 (66.4)	8 (61.5)	0.598
Statin	132 (71.4)	7 (63.6)	37 (68.5)	79 (73.8)	9 (69.2)	0.612

Data are presented as mean ± standard deviation or n (%). *p*-values from one-way ANOVA for continuous variables and chi-square test for categorical variables.

**Table 2:** Adverse events and safety outcomes during 5-year follow-up.

Adverse Events	Total Cohort (n = 185)	Canagliflozin (n = 11)	Dapagliflozin (n = 54)	Empagliflozin (n = 107)	*p*-Value
Common adverse events, n (%)					
Genital mycotic infections	16 (8.6)	1 (9.1)	4 (7.4)	10 (9.3)	0.912
- Female	13/104 (12.5)	1/7 (14.3)	3/30 (10.0)	8/64 (12.5)	0.891
- Male	3/81 (3.7)	0/4 (0.0)	1/24 (4.2)	2/43 (4.7)	0.854
Urinary tract infections	21 (11.4)	1 (9.1)	6 (11.1)	13 (12.1)	0.932
Volume depletion symptoms †	9 (4.9)	0 (0.0)	2 (3.7)	6 (5.6)	0.651
Serious adverse events, n (%)					
Diabetic ketoacidosis	0 (0.0)	0 (0.0)	0 (0.0)	0 (0.0)	-
Severe hypoglycemia ‡	0 (0.0)	0 (0.0)	0 (0.0)	0 (0.0)	-
Acute kidney injury	2 (1.1)	0 (0.0)	1 (1.9)	1 (0.9)	0.821
Bone fracture	3 (1.6)	0 (0.0)	1 (1.9)	2 (1.9)	0.912
Treatment discontinuation, n (%)	7 (3.8)	0 (0.0)	2 (3.7)	4 (3.7)	0.854
- Cost/insurance coverage	5 (2.7)	0 (0.0)	1 (1.9)	3 (2.8)	-
- Adverse event	2 (1.1)	0 (0.0)	1 (1.9)	1 (0.9)	-

† Volume depletion symptoms included dizziness, orthostatic hypotension, or syncope ‡ Severe hypoglycemia defined as requiring assistance from another person or resulting in hospitalization.

## Data Availability

The datasets used and/or analyzed during the current study are available from the corresponding author on reasonable request, subject to institutional data sharing policies.

## Supplementary Materials

The following supporting information can be downloaded at https://www.mdpi.com/article/10.3390/jcm14186365/s1, Supplementary Table S1. Baseline characteristics and outcomes for patients who switched between SGLT2 inhibitors during follow-up (n = 13). Supplementary Table S2. Comparison of baseline characteristics between patients with complete 5-year follow-up versus those lost to follow-up.

## Abbreviations

ACC: American College of Cardiology; AHA: American Heart Association; ASCVD: Atherosclerotic cardiovascular disease; BMI: Body mass index; CI: Confidence interval; CKD: Chronic kidney disease; CKD-EPI: Chronic Kidney Disease Epidemiology Collaboration; CVD: Cardiovascular disease; eGFR: Estimated glomerular filtration rate; HbA1c: Glycated hemoglobin; HDL: High-density lipoprotein; LDL: Low-density lipoprotein; MACE: Major adverse cardiovascular events; MENA: Middle East and North Africa; SAE: Serious adverse event; SD: Standard deviation; SGLT2i: Sodium-glucose cotransporter-2 inhibitors; T2DM: Type 2 diabetes mellitus; UAE: United Arab Emirates.
