# Supplementary material for: Long-Term Cardiorenal Benefits of Sodium-Glucose Cotransporter-2 Inhibitors in Patients with Type 2 Diabetes Mellitus: A Real-World Single-Center Experience
Source: J Clin Med. 2025 Sep 9;14(18):6365. doi: 10.3390/jcm14186365 (PMC12470307; doi:10.3390/jcm14186365)
Supplement: Supplementary file 1 [file jcm-14-06365-s001.zip › jcm-3831930-supplementary.pdf]

Supplementary Materials

Supplementary Table S1. Baseline characteristics and outcomes for patients who switched between SGLT2 inhibitors during follow-up (n=13)

**Characteristics**	**Switchers (n=13)**	**Overall Cohort (n=185)**	**p-value**
**Baseline Demographics**			
Age (years), mean ± SD	56.5 ± 10.2	57.0 ± 11.8	0.874
Female, n (%)	3 (23.1)	104 (56.2)	0.021*
Diabetes duration (years), mean ± SD	8.0 ± 5.1	8.2 ± 5.3	0.892
**Baseline Clinical Parameters**			
Weight (kg), mean ± SD	85.1 ± 17.9	86.4 ± 18.2	0.798
BMI (kg/m²), mean ± SD	32.8 ± 6.2	33.1 ± 6.7	0.871
Systolic BP (mmHg), mean ± SD	132.1 ± 15.8	132.5 ± 16.2	0.928
Diastolic BP (mmHg), mean ± SD	78.0 ± 9.5	78.3 ± 9.8	0.912
**Baseline Laboratory Values**			
HbA1c (%), mean ± SD	8.8 ± 1.7	8.7 ± 1.8	0.842
eGFR (mL/min/1.73m²), mean ± SD	91.2 ± 21.8	92.3 ± 22.1	0.858
Total cholesterol (mmol/L), mean ± SD	4.5 ± 1.1	4.5 ± 1.2	1.000
**Switching Patterns**			
Initial drug, n (%)			
- Empagliflozin	7 (53.8)	-	-
- Dapagliflozin	4 (30.8)	-	-
- Canagliflozin	2 (15.4)	-	-
Time to switch (months), median (IQR)	18 (12-28)	-	-
Reason for switching, n (%)			
- Inadequate glycemic control	6 (46.2)	-	-
- Cost/insurance coverage	4 (30.8)	-	-
- Minor adverse events	2 (15.4)	-	-
- Patient preference	1 (7.6)	-	-
**Outcomes at 12 months**			
HbA1c (%), mean ± SD	8.3 ± 1.8	8.2 ± 1.9	0.848
eGFR (mL/min/1.73m²), mean ± SD	92.0 ± 22.3	93.1 ± 21.9	0.856
Weight (kg), mean ± SD	84.0 ± 17.5	85.1 ± 17.9	0.825
BMI (kg/m²), mean ± SD	32.4 ± 6.1	32.7 ± 6.8	0.870
**Outcomes at 5 years**	**(n=9, 69.2%)**	**(n=120, 64.9%)**	
HbA1c (%), mean ± SD	8.3 ± 1.8	8.2 ± 1.8	0.865
eGFR (mL/min/1.73m²), mean ± SD	86.5 ± 23.2	87.0 ± 23.8	0.948
Weight (kg), mean ± SD	83.8 ± 17.2	84.8 ± 18.1	0.868

**Characteristics**	**Switchers (n=13)**	**Overall Cohort (n=185)**	**p-value**
BMI (kg/m²), mean ± SD	32.3 ± 6.0	32.5 ± 6.9	0.928
Change in HbA1c from baseline (%), mean ± SD	-0.5 ± 1.3	-0.5 ± 1.4	1.000
Annual eGFR decline (mL/min/1.73m²/year)	0.9	1.1	0.742
**Safety Outcomes, n (%)**			
Genital mycotic infections	1 (7.7)	16 (8.6)	0.906
Urinary tract infections	2 (15.4)	21 (11.4)	0.654
Serious adverse events	0 (0.0)	5 (2.7)	0.531
Permanent discontinuation	1 (7.7)	7 (3.8)	0.468

Notes: Data presented as mean ± standard deviation, median (interquartile range), or n (%) as appropriate. P-values from independent t-test for continuous variables and chi-square or Fisher's exact test for categorical variables comparing switchers to overall cohort.

Abbreviations: SD, standard deviation; BMI, body mass index; BP, blood pressure; HbA1c, glycated hemoglobin; eGFR, estimated glomerular filtration rate; IQR, interquartile range

Supplementary Table S2. Comparison of baseline characteristics between patients with complete 5-year follow-up versus those lost to follow-up

Characteristics	Complete 5-year follow-up (n=120)	Lost to follow-up (n=65)	p-value
**Demographics**			
Age (years), mean ± SD	57.3 ± 11.5	56.4 ± 12.3	0.619
Female, n (%)	69 (57.5)	35 (53.8)	0.633
UAE nationals, n (%)	98 (81.7)	51 (78.5)	0.598
Diabetes duration (years), mean ± SD	8.3 ± 5.2	8.0 ± 5.5	0.714
**Clinical Parameters**			
Weight (kg), mean ± SD	86.8 ± 18.0	85.6 ± 18.6	0.665
BMI (kg/m²), mean ± SD	33.2 ± 6.6	32.9 ± 6.9	0.768
Systolic BP (mmHg), mean ± SD	132.8 ± 16.0	131.9 ± 16.6	0.715
Diastolic BP (mmHg), mean ± SD	78.5 ± 9.7	77.9 ± 10.0	0.689
**Laboratory Values**			
HbA1c (%), mean ± SD	8.6 ± 1.8	8.8 ± 1.9	0.474
FPG (mmol/L), mean ± SD	9.7 ± 3.1	10.0 ± 3.4	0.541
eGFR (mL/min/1.73m²), mean ± SD	92.8 ± 21.8	91.4 ± 22.6	0.681
Total cholesterol (mmol/L), mean ± SD	4.5 ± 1.2	4.6 ± 1.2	0.583
HDL cholesterol (mmol/L), mean ± SD	1.1 ± 0.3	1.1 ± 0.3	0.894
LDL cholesterol (mmol/L), mean ± SD	2.6 ± 0.9	2.7 ± 0.9	0.468
Triglycerides (mmol/L), mean ± SD	1.8 ± 1.0	1.9 ± 1.0	0.512
UACR (mg/g), median (IQR)	28.5 (12.0-68.5)	31.2 (14.5-72.8)	0.642
**Comorbidities, n (%)**			
Hypertension	83 (69.2)	43 (66.2)	0.678
Dyslipidemia	94 (78.3)	49 (75.4)	0.648
Current smoker	12 (10.0)	7 (10.8)	0.868
Known CAD	15 (12.5)	8 (12.3)	0.970
Prior stroke/TIA	5 (4.2)	3 (4.6)	0.883
Heart failure	4 (3.3)	2 (3.1)	0.924
Peripheral arterial disease	3 (2.5)	2 (3.1)	0.813
**SGLT2i Distribution, n (%)**			
Empagliflozin	68 (56.7)	39 (60.0)	0.662
Dapagliflozin	36 (30.0)	18 (27.7)	0.741
Canagliflozin	7 (5.8)	4 (6.2)	0.932

Characteristics	Complete 5-year follow-up (n=120)	Lost to follow-up (n=65)	p-value
Switchers	9 (7.5)	4 (6.1)	0.731
**Concomitant Medications, n (%)**			
Metformin	112 (93.3)	60 (92.3)	0.793
Sulfonylurea	43 (35.8)	25 (38.5)	0.722
DPP-4 inhibitor	27 (22.5)	16 (24.6)	0.744
GLP-1 RA	8 (6.7)	4 (6.2)	0.890
ACE-I/ARB	78 (65.0)	40 (61.5)	0.641
Beta-blocker	42 (35.0)	21 (32.3)	0.714
Statin	87 (72.5)	45 (69.2)	0.638
Antiplatelet	59 (49.2)	30 (46.2)	0.696
**Reasons for Loss to Follow-up**			
Moved/relocated	-	28 (43.1)	-
Changed healthcare provider	-	18 (27.7)	-
Lost to follow-up (unknown reason)	-	12 (18.5)	-
Death (non-study drug related)†	-	4 (6.2)	-
Withdrew consent	-	3 (4.6)	-

Notes: Data presented as mean ± standard deviation, median (interquartile range), or n (%) as appropriate. P-values from independent t-test for continuous variables and chi-square or Fisher's exact test for categorical variables.

†Deaths were due to: COVID-19 complications (n=2), motor vehicle accident (n=1), and malignancy diagnosed after study enrollment (n=1). None were considered related to SGLT2i therapy.

Abbreviations: SD, standard deviation; BMI, body mass index; BP, blood pressure; HbA1c, glycated hemoglobin; FPG, fasting plasma glucose; eGFR, estimated glomerular filtration rate; HDL, high-density lipoprotein; LDL, low-density lipoprotein; UACR, urine albumin-creatinine ratio; IQR, interquartile range; CAD, coronary artery disease; TIA, transient ischemic attack; DPP-4, dipeptidyl peptidase-4; GLP-1 RA, glucagon-like peptide-1 receptor agonist; ACE-I, angiotensin-converting enzyme inhibitor; ARB, angiotensin receptor blocker
